# Shrinkage of Nasal Mucosa and Cartilage During Formalin Fixation

**DOI:** 10.4274/balkanmedj.2015.1470

**Published:** 2017-09-29

**Authors:** Leyla Kansu, Erdinç Aydın, Hampar Akkaya, Suat Avcı, Nalan Akalın

**Affiliations:** 1 Departments of Otolaryngology-Head and Neck Surgery, Başkent University School of Medicine, Ankara, Turkey; 2 Departments of Pathology, Başkent University School of Medicine, Ankara, Turkey; 3 Departments of Biochemistry, Başkent University School of Medicine, Ankara, Turkey

**Keywords:** Sinonasal tumours, shrinkage, histology, formaldehyde, surgical margin

## Abstract

**Background:**

After resection, specimens are subjected to formalin fixation during histological processing. This procedure can result in tissue shrinkage, with the amount of shrinkage related to tissue composition and tissue type.

**Aims:**

To evaluate the shrinkage of nasal mucosa and cartilage tissue and compare differences in shrinkage after resection, after formalin fixation, and during microscopic examination to understand differences in the rate of shrinkage of different tissue types.

**Study Design:**

Animal experimentation.

**Methods:**

Fresh nasal septa were excised from sheep (10 mm diameter in 40 sheep and 20 mm diameter in 40 sheep). The mucosa was separated from one side of the cartilage, with the contralateral mucosa remaining attached to the cartilage. Specimen diameters were measured in situ, after resection, after fixation for 6 or 24 hours (10% formalin), and during microscopic examination.

**Results:**

There were no differences between the in situ and after resection diameters of any tissue components (free mucosa, mucosa attached to cartilage, and cartilage) of all nasal specimens (10- or 20-mm diameter and 6- or 24-hour fixation). However, significant shrinkage occurred between resection and after-fixation. Regarding tissue specimens that were fixed for different durations (6 or 24 hours), we observed a significantly smaller mean tissue diameter in specimens fixed for 24 hours versus those fixed for 6 hours for mucosa attached to cartilage (in the 10-mm diameter after-fixation samples), free mucosa (in the 20-mm diameter after-fixation samples), mucosa attached to cartilage (in the 20-mm diameter after-fixation and microscopic measurement samples), and cartilage (in the 20-mm diameter after-fixation samples). Tissue shrinkage was greatest in free mucosal tissue and least in cartilage.

**Conclusion:**

These results should be considered when evaluating patients undergoing surgical procedures for nasal cavity and paranasal sinus malignancies. Surgical margins should be measured before fixation or evaluated if possible before fixation and shrinkage.

Carcinomas of the nasal cavity and paranasal sinuses (NCPS) account for 1% of all malignancies and 3% of head and neck malignancies. These neoplasms may be epithelial, mesenchymal, neural, neuroectodermal, or haematopoietic. Squamous cell carcinoma (SCC) is the most common malignancy of the mucosal surfaces of NCPS (1,2). The primary treatment of carcinoma of NCPS is complete surgical resection, which is usually followed by postoperative radiotherapy (3). The goal of surgical treatment is complete eradication of the primary tumour with a safe margin (4). Despite improvements in surgical techniques and radiotherapy, patients with SCC of NCPS have a poor prognosis, and the 5-year survival rate is 50% (1).

The most important prognostic factor for SCC of NCPS is complete surgical removal of the neoplasm. Failure to eradicate the primary tumour is the leading cause of local recurrence of this type of cancer. Local recurrence is likely when gross tumour remains, and this can lead to patient death. The presence of microscopic cancer at the margin of resection is associated with local recurrence and poor survival. Local recurrence occurs in approximately 50% of patients, even when surgical margins are microscopically negative for residual tumour (3).

Surgical and pathological margins may differ. The head and neck surgeon may determine the margin of normal tissue around the resected tumour 1 to 2 cm wide, but the margin measured by the pathologist may be smaller. This difference may be related to tissue shrinkage after resection and during specimen preparation (5). Most tissues shrink when placed in a formalin fixative solution (4,6). A formaldehyde solution is the most commonly used fixative during histopathological examination. However, this fixative may cause marked deformation of tissue dimensions and shape (5); therefore, pathologists typically report that resection margins closer than those measured by the surgeon during surgery (7).

A correction factor may be used to compensate for tissue shrinkage during specimen processing. The purpose of using a correction factor is to estimate the actual length of unfixed tumour in vivo and to obtain data that are comparable between laboratories (8). The composition and type of tissue may affect the amount of tumour shrinkage. Therefore, studies on changes in tissue size due to formalin fixation should be organ specific (9).

Studies on the shrinkage behaviour of tissue have been performed in liver, muscle, spleen, kidney, lingual mucosa, prostate, lung, cornea, colon, oesophagus, and brain tissue (6,7,10,11,12). For skin tissue, the degree of shrinkage caused by formalin fixation is controversial (13,14). A literature review revealed no previous reports on shrinkage of the nasal mucosa and cartilage of the nasal septum after excision and histological processing.

In the present study, our aim was to evaluate and quantify the shrinkage of specimens taken from the nasal mucosa and cartilage of sheep and to document whether inconsistencies exist between measurements of in situ margins before excision and histological margins.

## MATERIALS AND METHODS

### Animals

This experimental study included 80 heads from freshly killed sheep obtained from a local abattoir. The study was approved by our Institutional Review Board (Başkent University project no: DA10/22) and supported by research funds from our university.

### Procedure

**FIG. 1.**
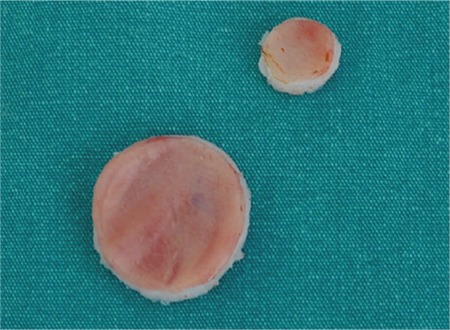
Nasal septa (full layer: mucosa, cartilage, and contralateral mucosa) were excised in two round diameters (10 and 20 mm).

**FIG. 2.**
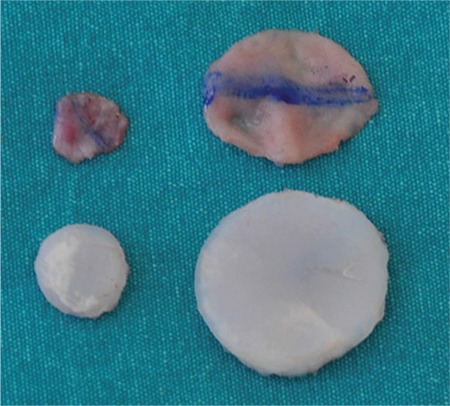
The mucosa from one side of the septum was dissected free, and the contralateral mucosa remained attached to the cartilage. The diameters of the free mucosa, mucosa remaining attached to the cartilage, and cartilage were measured with a millimetre ruler.

The nasal septa were removed from all sheep heads by the same surgeon (L.K.). Nasal septa that had lacerations or abnormal colour were excluded. Nasal septa (full layer: mucosa, cartilage, and contralateral mucosa) were excised in round diameters that were measured in situ and marked with a surgical marker (Devon surgical skin marker; Covidien, Minneapolis, MN, USA). Two in situ diameters were obtained: 10 mm (40 sheep) and 20 mm (40 sheep) (Figure 1). After excision, the mucosa from one side of the septum was dissected free, with the contralateral mucosa remaining attached to the cartilage. The diameters of the free mucosa, mucosa remaining attached to the cartilage, and cartilage were measured with a millimetre ruler (“after resection” diameters) (Figure 2). The free mucosa became contracted because it was very thin, but it was spread out on a hard, smooth surface before measurement.

All specimens were completely immersed in 10% neutral-buffered formalin (formaldehyde 37-40%; Merck, Darmstadt, Germany) immediately after excision, with measurements of length and calculations of percent differences also made immediately. After fixation for 6 or 24 hours, the specimens were removed from the formalin. Specimen diameters were measured by a pathologist (H.A.) (“after fixation” diameters). The tissue was marked, and sections were cut for paraffin embedding and histological preparation. One slide was prepared from each specimen, which was stained with haematoxylin and eosin. The diameters on the stained slide were measured with an ocular micrometer (“microscopic” diameters) (Axioscop 2; Carl Zeiss, Oberkochen, Germany).

The specimens were grouped with specimens of similar initial diameter (10 mm or 20 mm) and fixation time (6 hours or 24 hours), and the components of each specimen group were grouped separately (free mucosa, mucosa attached to cartilage, and cartilage).

### Statistical analysis

Data were analysed using statistical software (Statistical Package for the Social Sciences, version 22.0, SPSS Inc., Armonk, IBM Corp., NY, USA). Data are expressed as number (percent) or mean ± standard deviation. Comparisons were made by the Friedman test and Wilcoxon signed rank test with Bonferroni correction. The mean difference was significant at the p<0.017 level (p/n=0.05/3).

## RESULTS

There were no differences between the in situ and after-resection diameters of any tissue components (free mucosa, mucosa attached to cartilage, and cartilage) of all nasal specimens (10- or 20-mm diameter and 6- or 24-hour fixation). Therefore, no shrinkage had occurred. However, significant shrinkage occurred between resection and after-fixation (Table 1). Regarding tissue specimens that were fixed for different durations (6 or 24 hours), we observed a significantly smaller mean tissue diameter in specimens fixed for 24 hours versus those fixed for 6 hours for mucosa attached to cartilage (in the 10-mm diameter after-fixation samples), free mucosa (in the 20-mm diameter after-fixation samples), mucosa attached to cartilage (in the 20-mm diameter after-fixation and microscopic measurement samples), and cartilage (in the 20-mm diameter after-fixation samples) (Table 2).

## DISCUSSION

The present data confirmed that fixation in formalin causes significant shrinkage of nasal septal tissue, including free mucosa, mucosa attached to cartilage, and cartilage. The shrinkage occurred after fixation for 6 or 24 hours, and some tissue shrinkage was greater after a longer duration of fixation (Table 1, 2).

In patients with head and neck cancer, residual microscopic disease is associated with more frequent recurrence and shorter overall survival (15). Postoperative treatment options are affected by the presence of a tumour-free margin (16). Patients who have tumour present at surgical margins are offered additional surgery, and patients who have tumour near the resection margins are treated with additional surgery or external beam radiotherapy. The status of the surgical margin is the strongest predictor of local recurrence (17). Most patients (75%) who have tumour at the surgical margin may develop local recurrence or may have residual disease noted at reoperation (18). Measurements of the proximity of resected tumour to the tumour margins may be affected by tissue shrinkage, and this may be a factor in treatment recommendations and prognosis.

Surgical margins may be categorized as (1) clear (no microscopic carcinoma or dysplastic epithelium within 5 mm of the margin), (2) close (microscopic carcinoma or dysplastic epithelium within 5 mm of the margin but not at the margin), (3) dysplasia (dysplastic epithelium at the superficial margin but no carcinoma within 5 mm of the margin), or (4) involved (carcinoma at the margin in an intraoperative frozen section or postoperative pathological assessment). In head and neck cancer, the 5-year frequency of local control varies with margin status (clear, 91%; close, 80%; dysplasia, 82%; involved, 44%) (4).

The rate of local recurrence of head and neck carcinoma is greater with close margins (29%) than with clear margins (13%) (19), with a poor prognosis in patients with persistent or recurrent local or regional disease (20); the frequency of local recurrence is determined by margin status (55% local recurrence rate for microinvasive, 50% for in situ, and 45% for close margins) (17). Therefore, it is important that surgeons make every possible effort to achieve clear margins.

Tumours of NCPS are challenging because of the complex three-dimensional anatomy that may complicate the in situ measurement of proposed margins. Furthermore, the mucosa and underlying tissues (cartilage and bone) may vary in functioning as barriers to tumour spread (5). In addition, the present study shows that tissue shrinkage after tissue resection may confound the accurate measurement of margin size (Table 1, 2).

Surgeons attempt to produce clear margins with wide local excisions. However, margin distances may be markedly smaller in the pathology report versus in situ measurements made before excision. This may occur because of tissue shrinkage during histological processing. The amount of shrinkage may be affected by tissue composition (content of water, fat, or connective tissue) and histological procedure; however, the quantitative effect of these factors is not known. Therefore, studies that evaluate changes in tissue size should be specific to different tissue types and composition (21,22).

Phosphate-buffered formaldehyde is the most commonly used fixative for light microscopy because of its low cost, high ease of preparation, and good preservation of morphological details with few artifacts (23,24,25). However, this fixative may cause marked deformation of tissue geometry (dimension and shape) (12). The amount of shrinkage caused by formalin fixation may depend on specimen type and laboratory-specific circumstances such as formalin concentration and duration of fixation (8). For example, canine laryngeal samples have been shown to develop marked tissue deformation and distortion as a result of formalin fixation and other histological procedures (12). Marked changes in tissue weight (34%) have also been observed in other organs after formalin fixation (22).

Tissue specimens are affected by formalin fixation in two phases. First, the fixative penetrates the tissue by diffusion and accumulates in the tissue. Second, formalin has a gelling action, chemically binding to protein amino groups and causing extensive cross-links between proteins and nucleic acids. Although formalin may stabilize and preserve tissue ultrastructure, it may cause histological changes such as distortion, vacuolization, and cell shrinkage. These effects may change tissue immunohistochemical reactivity and cause gross changes in specimen shape and size. These changes may cause discrepancies between measurements of tumour size made by surgeons versus pathologists (9). Specimen shrinkage caused by fixation may cause the margins to appear closely involved with tumour and may change the margin classification from negative to positive.

Tissue shrinkage associated with formalin fixation during histological processing is a major concern in various tissues and organs. Margins in colorectal and oesophageal specimens may shrink to half the original size. Formalin fixation may decrease the length of the upper (32%) and lower (39%) margins of the oesophagus (7). In human brain tissue, marked shrinkage of tissue volume (48%) and length (20%) may occur despite different concentrations of formalin (26). Formalin fixation may cause longitudinal shrinkage in the brainstem (1% to 8%) and may increase the weight and size of the cerebrum and cerebellum (10). Cervical tissue has been shown to undergo marked shrinkage (linear, 15%) caused by 8% formalin fixation, alcohol dehydration, and paraffin embedding (27). After 24 hours in 8% formalin fixative, cervical tissue was shown to shrink longitudinally (3%) and transversely (2.4%), with prolonged fixation in formaldehyde causing more shrinkage and hardening of tissue (27). In bovine kidney and liver tissue, histological processing with alcohol dehydration and paraffin infiltration may cause marked shrinkage (tissue length changed by 8% to 17%) (28). Liver tissue may shrink (<10%) after formalin fixation (6). In lung tissue, formalin fixation may cause linear tissue shrinkage (24% to 36%) (21). Blood vessel diameter may decrease by 14% in buffered formalin and an additional 16% following histological processing, embedding in paraffin, and staining, with canine arterial rings fixed with formalin and embedded in paraffin showing marked shrinkage in cross-sectional area (19%) (29). In the canine oral cavity, 10% formalin fixation, paraffin embedding, and staining caused marked shrinkage of tongue surface mucosal tissue (31%), deep tongue muscle (35%), and labiobuccal mucosal tissue (47%) (5). In the oral cavity, shrinkage of tongue (25%) and buccal specimens (33%) has been noted after resection and fixation (5,15). Vocal fold thickness has also been shown to decrease (9% to 24%) after formalin fixation and additionally (0% to 14%) after histological processing (12). In breast tissue, specimen margins decreased an average of 3.5 mm (34%) after fixation (16). Prostatectomy specimens may also decrease in length (4.1%) after fixation (30). In the present study, we observed shrinkage in nasal septal tissue samples after fixation (Table 1, 2).

In clinical practice, most laboratories process biopsy specimens on the day of arrival. The penetration of formaldehyde fixative is rapid, and approximately half-maximum formaldehyde binding is achieved within 100 minutes of tissue immersion in the fixative (23). The optimal period of specimen fixation necessitates sufficient penetration of the fixative and avoidance of secondary shrinkage or excessive tissue hardening (16). Formalin penetrates tissue by approximately 2.5 mm in 4 hours (24,29). In prostatectomy specimens fixed in 10% buffered formalin, tissue shrinkage was shown to be negligible during the first 2 hours of fixation, slight after 4 hours, and maximal by 12 to 14 hours, with no additional shrinkage at 24 and 48 hours (30).

In the present study, shrinkage varied between tissue types, with more tissue shrinkage observed in mucosal tissue than in cartilage (Table 1). Shrinkage was less in nasal mucosa that was attached to cartilage than in free mucosa because the attachments to cartilage helped maintain mucosal dimensions. The tissue with the least shrinkage was cartilage (Table 1). The present data may provide a rationale for decisions about adequate resection margins for carcinoma of NCPS. Accordingly, with the present results, the pathologist should alert those reading margins about the different shrinkage ratios for different tissue types.

There were no significant differences between fixation for 6 and 24 hours in the 10 mm free mucosa and cartilage samples. However, we observed significant differences between fixation for 6 hours and 24 hours in the 10 mm mucosa samples attached to cartilage (Table 2). This finding could not be explained by tissue structure and fixation time, and additional research regarding this finding may be necessary.

Greater shrinkage was observed in nasal mucosal tissue than in cartilage; this difference may strongly affect oncological results. These results should be considered during surgical margin resection. Although the extent of surgical resection cannot be determined only by possible tissue shrinkage, our study shows that such possibilities exist and both the surgeon and the pathologist should therefore be cautious during surgical margin determination. Measuring each surgical margin before fixation or evaluating margins before tissues are subjected to shrinkage may be appropriate.

In this study, sheep were used as an animal model. Although human nasal tissue is not similar to that of animals, our animal experiment may provide significant data and inspire further research. Further studies in human nasal tissue would be of interest and are warranted.

## Figures and Tables

**Table 1:**
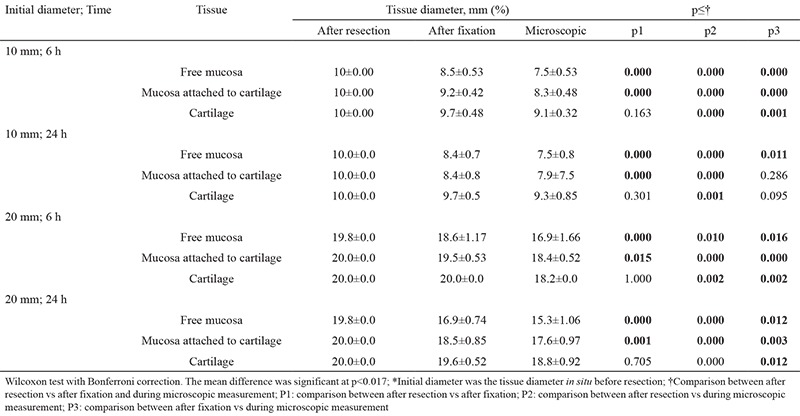
Relation between nasal septum tissue initial diameter, duration of fixation, and diameter after resection, after fixation, and during microscopic measurement*

**Table 2:**
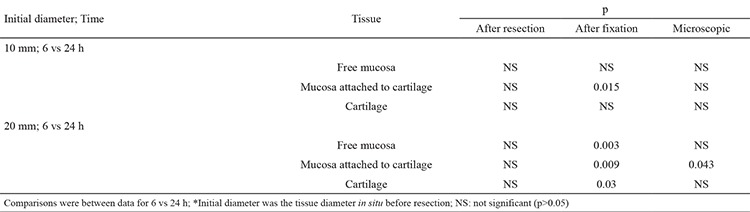
Relation between duration of fixation and tissue diameter for nasal septum tissue specimens*
